# In vivo assessment of the ocular biomechanical properties in patients with idiopathic normal pressure hydrocephalus

**DOI:** 10.1007/s10792-024-02922-3

**Published:** 2024-02-02

**Authors:** Nicola Valsecchi, Matilde Roda, Simone Febbraro, Eleonora Trolli, Giorgio Palandri, Giulia Giannini, David Milletti, Costantino Schiavi, Luigi Fontana

**Affiliations:** 1 Ophthalmology Unit, Dipartimento di Scienze Mediche e Chirurgiche, Alma Mater Studiorum, University of Bologna, Bologna, Italy; 2 IRCCS Azienda Ospedaliero-Universitaria di Bologna, Bologna, Italy; 3 Unit of Neurosurgery, IRCCS Istituto delle Scienze Neurologiche di Bologna, Bologna, Italy; 4 Department of Biomedical and Neuromotor Sciences (DIBINEM), IRCCS Istituto delle Scienze Neurologiche di Bologna, Bologna, Italy; 5 Unit of Rehabilitation Medicine, IRCCS Istituto delle Scienze Neurologiche di Bologna, Bologna, Italy

**Keywords:** Idiopathic normal pressure hydrocephalus, Ocular response analyzer, Ocular biomechanical properties, Open angle glaucoma

## Abstract

**Purpose:**

Idiopathic normal pressure hydrocephalus (iNPH) is associated with an increased prevalence of open-angle glaucoma, attributed to variations of the pressure gradient between intraocular and intracranial compartments at the level of the lamina cribrosa (LC). As ocular biomechanics influence the behavior of the LC, and a lower corneal hysteresis (CH) has been associated to a higher risk of glaucomatous optic nerve damage, in this study we compared ocular biomechanics of iNPH patients with healthy subjects.

**Methods:**

Twenty-four eyes of 24 non-shunted iNPH patients were prospectively recruited. Ocular biomechanical properties were investigated using the ocular response analyzer (Reichert Instruments) for the calculation of the CH, corneal resistance factor (CRF), Goldmann-correlated intraocular pressure (IOPg), and corneal-compensated intraocular pressure (IOPcc). Results were compared with those of 25 eyes of 25 healthy subjects.

**Results:**

In iNPH eyes, the median CH value and interquartile range (IQR) were 9.7 mmHg (7.8–10) and 10.6 mmHg (9.3–11.3) in healthy controls (*p* = 0.015). No significant differences were found in IOPcc [18.1 mmHg (14.72–19.92) vs. 16.4 mmHg (13.05–19.6)], IOPg [15.4 mmHg (12.82–19.7) vs. 15.3 mmHg (12.55–17.35)], and CRF [9.65 mmHg (8.07–11.65) vs. 10.3 mmHg (9.3–11.5)] between iNPH patients and controls.

**Conclusions:**

In iNPH patients, the CH was significantly lower compared to healthy subjects. This result suggests that ocular biomechanical properties may potentially contribute to the risk of development of glaucomatous optic nerve damage in iNPH patients.

## Purpose

Idiopathic normal pressure hydrocephalus (iNPH) is a chronic neurological syndrome characterized by progressive onset of gait dysfunction, cognitive impairment, and urinary incontinence [1]. iNPH is a disease of the elderly population, and its hallmark is the enlargement of the intracerebral ventricles associated with normal intracranial pressure (ICP) with no visible obstruction to cerebrospinal fluid (CSF) flow. The exact pathophysiological mechanism in iNPH is still controversial, and reduction in CSF drainage, neuroinflammation, vascular hypoperfusion, and impaired glymphatic circulation are thought to play a role in the disease development [2, 3]. Clinical and neuroradiological criteria helps in distinguishing iNPH from “hydrocephalus ex vacuo” secondary to brain atrophy. [4, 5]

Furthermore, iNPH is one of the few causes of dementia potentially reversible by means of ventricular shunting, a procedure that is associated with an ICP lowering of 3 mm Hg on average, with a success rate of up to 85% at 12 months [6–8]. In recent years, several studies reported an ophthalmological involvement in both non-shunted and shunted iNPH patients, with a higher prevalence of Open Angle Glaucoma (OAG) compared to the general population. [9–16]. The increased risk of glaucomatous damages is thought to be secondary to changes in the pressure gradient between intraocular and intracranial compartments occurring at the lamina cribrosa level, the mesh-like membrane present in the sclera where retinal ganglion cell axons exit from the eye. The trans-laminar cribrosa pressure gradient (TCPG) is the difference between the intraocular pressure (IOP) and intracranial pressure (ICP) across the lamina cribrosa. An increased TCPG determines a compression of the retinal ganglion cell axons that pass through the lamina cribrosa pores, with progressive damage and cellular loss. [17, 18] The behavior of the LC is influenced by the biomechanical properties of the eye, as an increased stiffness of the LC and the peripapillary sclera may result in reduced compliance at the optic nerve head, with a higher susceptibility to IOP-induced glaucomatous injury. [19] Currently, corneal biomechanics can be assessed in vivo using either the Ocular Response Analyzer (ORA) (Reichert Instruments, Depew, New York) or the CorVis ST (Radan, Sanaa, Yemen). The Ocular Response Analyzer (ORA) is a non-contact tonometer that provides the measure of two parameters of corneal biomechanics: corneal resistant factor (CRF) that reflects the maximum correlation with central corneal thickness, and corneal hysteresis (CH) that measures the viscoelastic response of the cornea. Previous studies reported that eyes with lower CH had faster rates of visual field loss and progressive optic nerve damage than those with higher CH, suggesting that CH may serve as a surrogate biomarker of the viscoelastic properties of the LC, and lower CH may indicate a decreased ability of the posterior tissues to compensate for IOP changes. [20–22]

The purpose of this study was to compare the ocular biomechanical properties of non-shunted iNPH eyes to those of age-matched controls using the ORA, in order to assess if iNPH patients may have an increased risk of glaucomatous optic nerve damage.

## Methods

### Study population

Twenty-four iNPH patients were recruited between November 2021 and February 2023 from a prospective longitudinal cohort study carried out at the Unit of Ophthalmology, IRCSS University of Bologna, in collaboration with the Bologna PRO-HYDRO study group of the Istituto delle Scienze Neurologiche di Bologna (ISNB). The study was adherent to the tenets of the Declaration of Helsinki and was approved by the Institutional Review Board/Ethics Committee of the local health service of Bologna, Italy (Cod CE: 809/2021). Written informed consent was obtained from all subjects included in the study.

The multidisciplinary Bologna PRO-HYDRO study group discussed patients with a suspected diagnosis of iNPH. [23] Patients included in this study were only those who fulfilled the diagnostic clinical and neuroradiological criteria for “probable” iNPH according to the guidelines published by Relkin et al. and were considered eligible for ventriculo-peritoneal (VP) shunt surgery. [4]

The objective of the study was to evaluate corneal biomechanics using the ORA device (Reichert Instruments, Depew, New York) in the eyes of patients with iNPH and compare the results with the eyes of healthy age-matched controls.

We excluded INPH patients with an ophthalmic history of ocular trauma, retinal detachment, corneal opacities, advanced cataract, age-related macular degeneration, diabetic retinopathy, high myopia <  − 6 diopters or hypertropia >  + 3 diopters, axial length (AL) > 26 mm and < 21 mm, history of uveitis, and pachymetry below 450 μm and greater than 650 μm. Also, we excluded eyes with a previous diagnosis of glaucoma or ocular hypertension, and eyes that received previous laser treatment or topical antiglaucoma treatment.

The control group consisted of twenty-five healthy subjects of age comprised between 65 and 85 years, selected according to the same exclusion criteria described for the iNPH group.

### Clinical assessment

Each iNPH patient underwent a comprehensive ophthalmological eye examination, including best-corrected visual acuity (BCVA) assessment reported in logarithm of the minimum angle of resolution (LogMar), refraction assessment reported as spherical equivalent (SE), slit lamp examination of the anterior segment, indirect ophthalmoscopy, axial length (AL) and central corneal thickness (CCT) measurements using the IOLMaster 700 (Carl Zeiss Meditec AG, Jena, Germany).

### Ocular response analyzer measurements

Briefly, the ORA generates a precisely metered air pulse that causes a dynamic bi-directional applanation process, during which two applanation pressure events are measured. During the first applanation pressure event (*A*1), an air puff pushes the cornea inwardly inducing a change from a convex to a concave shape (loading phase). When the cornea regains its baseline convex state (unloading or recovery phase), the second applanation pressure event (*A*2) occurs. The difference between the two applanation pressure values (*P*1 and *P*2, respectively) is referred as the CH. CRF value is instead obtained as a linear function of the two values. The Goldmann-correlated IOP (IOPg) is calculated as the average of two pressure values (*P*1 and *P*2, respectively), while the corneal-compensated IOP (IOPcc) represents the empirically determined IOP value that compensates for the effects of corneal biomechanics. (12, 14) All the ORA examinations were performed by two operators (N.V., S.F.), both masked to the subject’s characteristics. Before each examination, central corneal thickness (CCT) values obtained with an ultrasonic pachymeter (Dicon P55, Paradigm Medical Industries Inc., Salt Lake City, UT, USA) were inserted in the software. All measurements were obtained at the same day time between 10 am and 12 pm, to prevent the potential confounding effect of diurnal variation of IOP. All included ORA measurements had a waveform score > 7.0. (15) The best values of 4 measurements with desirable curves were used for the analysis. Whenever both eyes met the criteria, the right eye of each participant was included in the statistical analysis.

### Statistical analysis

Normality was tested with the Shapiro–Wilk test. As variables were not normally distributed, they were reported as median and interquartile ranges (IQR), and non-parametric tests were used in the statistical analysis. The Chi-square test was used to measure the association between two categorical variables.

The Mann–Whitney test was used to evaluate the differences in demographic characteristics, CH, CRF, IOPcc, IOPg, CCT, SE, and AL between the eyes of iNPH patients and healthy age-matched controls. *P* values < 0.05 were considered statistically significant. Statistical analysis was performed using IBM Statistical Package for Social Sciences version 26.

## Results

### Demographic characteristics

A total of 24 eyes of 24 iNPH patients and 25 eyes of 25 healthy individuals were included in the study. The median age of iNPH patients was 75.5 (IQR = 71.5–80) years old, and females were 29.2%. Four patients were pseudophakic, with a median SE of + 1 D (IQR =  − 1—+ 1), and a median BCVA of 0.1 logMAR (IQR = 0.2–0). The median AL and CCT were 24.15 mm and 531.5 μm, respectively. iNPH patients’ demographics were comparable to the control group (*p* > 0,05). Demographic data and clinical parameters are listed in Table 1 and 2**.**

**Table 1:** Demographic characteristics of the iNPH patients and age-matched healthy controls

Demographics	iNPH (*n* = 24)	Controls (*n* = 25)	*P* value
Females, number (%)	7 (29.2%)	10 (40%)	0,551
Age, median (IQR)	75.5 (71.5–80)	75 (69–81.5)	0,711
Pseudophakic, number (%)	4 (16.7%)	3 (12%)	0,702

*IQR* Interquartile range

**Table 2:** Clinical characteristics of the iNPH patients and age-matched healthy controls

Parameters	iNPH	Controls	*P* value
BCVA (LogMar)			
Median	0.1	0.1	0.634
Interquartile range	0.2–0	0.2–0	
Spherical equivalent (*D*)			0.728
Median	+ 1	+ 1	
Interquartile range	− 1 to + 1	− 1.25 to + 1.37	
CCT (μm)			0.450
Median	531.5	540	
Interquartile range	505–558	523–554	
AL (mm)			0.180
Median	24.15	23.56	
Interquartile range	22.91–24.85	22.78–24.23	

*BCVA* Best corrected visual acuity, *SE* Spherical equivalent, *CCT* Central corneal thickness, *AL* axial length

### Ocular biomechanical properties

In all patients, the waveform score was ≥ 7. The median value was 8 (IQR = 7.4–8.5) in the iNPH patients and 8.6 (IQR = 7.4–9.1) in the control group (*p* = 0.100).

CH was significantly lower in iNPH patients compared to healthy age-matched controls [median 9.7 mmHg (IQR = 7.82–10) vs. median 10.6 mmHg (IQR = 9.3–11.3 mmHg), respectively, *p* = 0.015)].

**Fig. 1:**
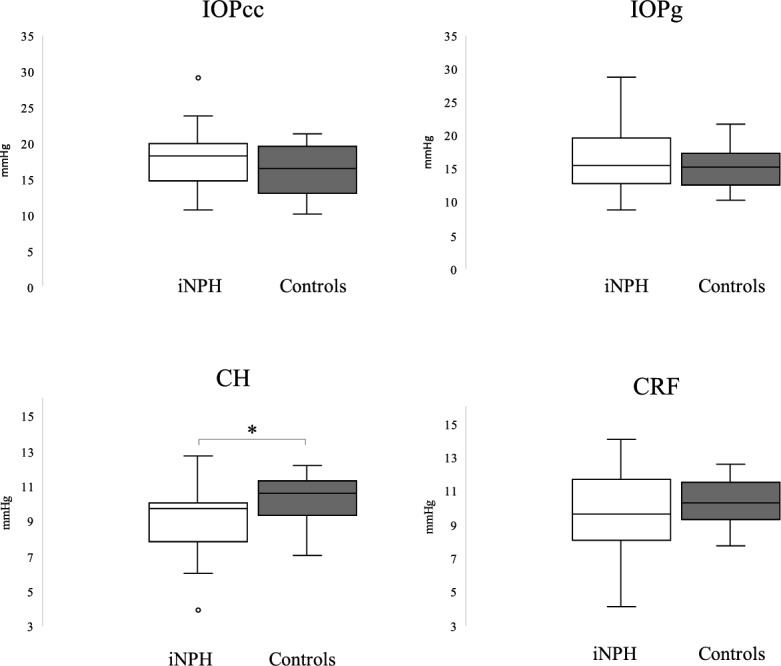
Ocular biomechanical parameters between non-shunted iNPH patients and age-matched controls are shown

We found no statistically significant differences between iNPH patients and controls in IOPcc [median 18.1 mmHg (IQR = 14.72–19.92] vs. median 16.4 mmHg (13.05–19.6), respectively, *p* = 0.150], IOPg [median 15.4 mmHg (IQR = 12.82–19.7) vs. median 15.3 mmHg (IQR = 12.55 vs. 17.35), respectively, *p* = 0.610], and CRF [median 9.65 mmHg (IQR = 8.07 – 11.65) vs. median 10.3 mmHg (IQR = 9.3–11.5), respectively, *p* = 0.412]. See Fig. 1.

## Discussion

In the present study, we investigated the biomechanical properties of the globe in patients with iNPH using the ORA. The main finding of the present study was that the CH was significantly lower in iNPH patients compared to controls (median 9, 7 vs. 10,6 mmHg, respectively, *p* = 0.028). On the other hand, we found no statistically significant differences in IOPcc, IOPg, and CRF between the two groups. CH is a biomechanical property that reflects the viscous damping ability of the globe when an external force is applied [24]. A higher CH reflects a better ability of the globe to absorb and dissipate energy, with increased flexibility. On the contrary, a lower CH reflects less flexibility, with a worse capacity for dissipating energy. There is speculation that eyes with lower CH might also have an optic disc that is more likely to suffer glaucomatous damage from raised IOP [25]. Previous studies showed that CH was lower in patients with OAG compared to healthy patients, suggesting that CH may serve as a surrogate biomarker of the viscoelastic properties of the LC, posterior sclera, and other optic nerve head structures [26]. Moreover, lower CH has been associated with an increased risk of progressive optic nerve damage and visual field defects compared to higher CH, even in patients with controlled values of IOP. [21, 27] In the present study, we observed reduced values of CH in eyes of non-glaucomatous non-shunted iNPH patients compared to healthy age-matched controls, suggesting that this population of patients may have a higher susceptibility to future glaucomatous damage.

Several studies reported an association between OAG and iNPH. Chang et al., reported a history of glaucoma in 18, 1% of 72 shunted and non-shunted patients with iNPH. [10] The higher prevalence of OAG in iNPH is thought to be secondary to modifications of the TCPG, defined as the difference between IOP and ICP across the lamina cribrosa.

In shunted iNPH patients, the increased risk of glaucomatous damages is thought to be secondary to a reduction in the ICP following shunt surgery, determining an increased TCPG and compression of the retinal ganglion cell axons that pass through the LC pores. Gallina et al., reported a prevalence of normal tension glaucoma (NTG) of 40.9% among 22 iNPH patients who underwent VP shunt placement. Moreover, they recently reported that 75% of INPH patients had been diagnosed with NTG within 10 years after VP shunt surgery. [9, 15]

On the other hand, a previous study by Igarashi et al., reported a prevalence of NTG of 55% in 20 non-shunted iNPH patients. [11] Moreover, a recent study by Eleftheriou et, al reported reduced values of ganglion cell complex (GCC) in non-shunted iNPH patients before and after the CSF tap test, and reduced values of retinal nerve fiber layer (RNFL) in non-shunted iNPH patients after the CSF tap test compared to healthy individuals. [12]

However, the exact pathophysiological mechanism responsible for the increased prevalence of OAG in non-shunted iNPH patients has not been fully understood. It has been postulated that the impaired flow of CSF described in iNPH may cause an accumulation of neurotoxins and diminished nutrition for retinal ganglion cell axons, leading to a progression of glaucomatous damage. [11] Moreover, in the first stage of iNPH, oscillations of high ICP wave amplitude have been observed, responsible for the expansion of the ventricles in the absence of an ICP increase. [28] These transient alterations of the ICP may be responsible for alterations in the TCPG, leading to progressive optic nerve damage. [11, 29]

In the present study, the reduced values of CH observed in iNPH patients may indicate a decreased ability of the LC to compensate for transient IOP and ICP changes, increasing the risk of progressive optic nerve damage compared to healthy individuals. To the best of our knowledge, this is the first study that investigated the ocular biomechanical properties in iNPH patients. Therefore, as the ocular biomechanics in iNPH patients are less likely to absorb the stress of increased pressure, IOP should be assessed routinely at the time of diagnosis and settled on lower values, to reduce the risk of glaucomatous damages. Moreover, as previously suggested by Gallina et al., it is essential to not exceed in decreasing the ICP values after the VP shunt surgery, to not determine an increased translaminar pressure responsible for the risk of glaucomatous damage after surgical intervention. [9]

However, future studies are necessary in order to confirm and better understand our findings, and to investigate the pathophysiological mechanism that could explain the reduced values of CH observed in iNPH patients.

The main strengths of the present study are the prospective design of the study and the strict inclusion criteria. We excluded iNPH patients with a diagnosis of OAG, patients under topical antiglaucoma treatment, or those who underwent previous laser treatment, as previous studies showed that these factors may influence the biomechanical properties of the globe. [30–34] Moreover, all measurements were obtained at the same day time between 10 am and 12 pm, to prevent the potential confounding effect of diurnal variation of IOP. [35]

The main limitation of the study is the relatively small cohort of patients included. Also, we included in the study only iNPH patients before shunt surgery. Future studies will be necessary to compare the ocular biomechanics after VP shunt, to assess if shunt surgery could influence the ocular biomechanical characteristics.

Also, further studies are needed to prospectively define the incidence and types of glaucomatous damages in both non-shunted and shunted iNPH patients, as the pathophysiological mechanisms implicated in the different forms of Glaucoma are different. Moreover, it would be interesting to assess the ocular biomechanical properties in glaucomatous iNPH patients and non-glaucomatous iNPH patients, in order to understand the exact role of CH in the progression of optic nerve damage in this population of patients.

## Conclusions

In the present study, we observed that iNPH patients presented a statistically significant lower CH compared to healthy age-matched individuals. This result suggests that ocular biomechanical properties may potentially contribute to the risk of development of glaucomatous optic nerve damage in iNPH patients. Further studies are needed in order to better understand the exact role of CH in the progression of optic nerve damage in iNPH patients.

## Data Availability

The datasets used and/or analyzed during the current study are available from the corresponding author upon reasonable request.

## Abbreviations

AL: Axial length
BCVA: Best-corrected visual acuity
CCT: Central corneal thickness
CH: Corneal hysteresis
CRF: Corneal resistance factor
CSF: Cerebrospinal fluid
GCC: Ganglion cell complex
ICP: Intracranial pressure
iNPH: Idiopathic normal pressure hydrocephalus
IOP: Intraocular pressure
IOPcc: Corneal-compensated intraocular pressure
IOPg: Goldmann-correlated intraocular pressure
IQR: Interquartile range
LC: Lamina cribrosa
NTG: Normal-tension glaucoma
OAG: Open angle glaucoma
ORA: Ocular response analyzer
RNFL: Retinal nerve fiber layer
SE: Spherical equivalent
TCPG: Trans-laminar cribrosa pressure gradient
VP: Ventriculo-peritoneal
